# Supplementary material for: Microbial material cycling, energetic constraints and ecosystem expansion in subsurface ecosystems
Source: Proc Biol Sci. 2020 Jul 29;287(1931):20200610. doi: 10.1098/rspb.2020.0610 (PMC7423649; doi:10.1098/rspb.2020.0610)
Supplement: Appendices (Supplemental Information) [file rspb20200610supp1.docx]

**Supporting Information**

**Appendix**

**Dynamic behaviour of Eq. (4)**

In Section S1, we explain the method of searching for a locally stable steady state of the dynamics (Eq. (4)). In this procedure, we exclude those that are obviously unstable. Subsequently, in Section S2, we show that we always have a single candidate for the stable-steady state. Finally, in Section S3, we prove that this candidate is, in fact, locally stable in dynamics (Eq. (4)).

**S1. Search for the locally stable steady state**

*Parameter reduction of Eqs. (4a–c)*

Let $\bar{k_{1}}=k_{1}B_{e}$, $\bar{k_{2}}=k_{2}C$, $\bar{r_{1}}=r_{1}\frac{B_{e}}{K_{1B_{e}}+B_{e}}$, $\bar{r_{2}}=r_{2}\frac{C}{K_{2C}+C}$, *f*_1_ = *q*_1_*c*_1_*r*_1_ and *f*_2_ = *q*_2_*c*_2_ *r*_2_, and dropping the bars, Eqs. (4a–c) are rewritten as follows:

$\frac{dA}{dt}={-k}_{1}A+k_{2}\left( A_{T}-A \right)-r_{1}\frac{A}{K_{1}+A}x_{1}+r_{2}\frac{A_{T}-A}{K_{2}+\left( A_{T}-A \right)}x_{2}$ , (S1a)

$\frac{dx_{1}}{dt}=\left( F_{1}(A)-m_{1}-{s_{1}x}_{1} \right)x_{1}$ , (S1b)

$\frac{dx_{2}}{dt}=\left( F_{2}(A)-m_{2}-{s_{2}x}_{2} \right)x_{2}$ , (S1c)

where

$F_{1}(A)=f_{1}\frac{A}{K_{1}+A}\left( \gamma_{1}+RTln\frac{A}{{(A}_{T}-A)} \right)$ where $\gamma_{1}=-\Delta_{r}{G^{\circ}}_{1}+RTln\frac{B_{e}}{B}$, (S2a)

$F_{2}(A)=f_{2}\frac{A_{T}-A}{K_{2}+\left( A_{T}-A \right)}\left( \gamma_{2}+RTln\frac{{(A}_{T}-A)}{A} \right)$ where $\gamma_{2}=-\Delta_{r}{G^{\circ}}_{2}+RTln\frac{C}{C_{e}}$. (S2b)

Let $\left( \hat{A},\hat{x}_{1},\hat{x}_{2} \right)$ be the stable equilibrium of the dynamics (S1) and (S2). We define

$\varphi_{1}=\frac{1}{s_{1}}\left( F_{1}(A)-m_{1} \right)$, if $F_{1}(A)>m_{1}$; $\varphi_{1}=0$, if $F_{1}(A)\leq m_{1}$. (S3a)

$\varphi_{2}=\frac{1}{s_{2}}\left( F_{2}(A)-m_{2} \right)$, if $F_{2}(A)>m_{2}$; $\varphi_{2}=0$, if $F_{2}(A)\leq m_{2}$. (S3b)

Then, from Eqs. (S1b) and (S1c), we have

$\hat{x}_{1}=\varphi_{1}\left( \hat{A} \right)$ and $\hat{x}_{2}=\varphi_{2}\left( \hat{A} \right)$. (S4)

Then, Eq. (1a) becomes

$0=-\hat{A}\left[ k_{1}+r_{1}\frac{\hat{A}}{K_{1}+\hat{A}}\varphi_{1}\left( \hat{A} \right) \right]+\left( A_{T}-\hat{A} \right)\left[ k_{2}+r_{2}\frac{A_{T}-\hat{A}}{K_{2}+\left( A_{T}-\hat{A} \right)}\varphi_{2}\left( \hat{A} \right) \right]$. (S5)

The first term of Eq. (S5) indicates the rate at which A is consumed, and the second term is the rate at which A is recovered. The balance between these two terms determines the final equilibrium abundance of A. The first term is an increasing function of $A,$ and the second term is a decreasing function of $A$. Once the equilibrium $A$ is determined from Eq. (S5), we can determine the abundance of the two types of microbe from Eq. (S4). Hence, there exists only a single equilibrium in this system.

According to the analysis in the later section, the equilibrium is always locally stable.

The effect of the recycling of the material between two types of microbe exists only when microbes of both types exist ($\hat{x}_{1}=\varphi_{1}\left( \hat{A} \right)>0$, and $\hat{x}_{2}=\varphi_{2}\left( \hat{A} \right)>0$). We can draw two curves for the two terms in Eq. (S5), as shown in Fig. 3. The equilibrium level of $\hat{A}$ is equal to the intersection of the two curves.

Let $A_{1}$ be the solution of $F_{1}(A_{1})=m_{1}$ and $A_{2}$ be the solution of $F_{2}(A_{2})=m_{2}$; both are between 0 and $A_{T}$. Then, we have the following property:

$\varphi_{1}\left( A \right)=0$, for $0<A<A_{1}$; and $\varphi_{1}\left( A \right)>0$, for $A_{1}<A<A_{T}$. (S6a)

$\varphi_{2}\left( A \right)>0$, for $0<A<A_{2}$; and $\varphi_{2}\left( A \right)=0$, for $A_{2}<A<A_{T}$. (S6b)

Combining these two, we have the following two situations:

If $A_{1}<A_{2}$, then

$\varphi_{1}\left( A \right)=0$ and $\varphi_{2}\left( A \right)>0$ for $0<A<A_{1}$. (S7a)

$\varphi_{1}\left( A \right)>0$ and $\varphi_{2}\left( A \right)>0$ for $A_{1}<A<A_{2}$. (S7b)

$\varphi_{1}\left( A \right)>0$ and $\varphi_{2}\left( A \right)=0$ for $A_{2}<A<A_{T}$. (S7c)

In this case, the stable coexistence of two types of microbe, and hence recycling at the equilibrium, is possible.

If $A_{2}<A_{1}$, then

$\varphi_{1}\left( A \right)=0$ and $\varphi_{2}\left( A \right)>0$ for $0<A<A_{12}$. (S8a)

$\varphi_{1}\left( A \right)=\varphi_{2}\left( A \right)=0$ for $A_{2}<A<A_{1}$. (S8b)

$\varphi_{1}\left( A \right)>0$ and $\varphi_{2}\left( A \right)=0$ for $A_{1}<A<A_{T}$. (S8c)

In the latter case, the stable coexistence of two types of microbe is impossible.

In the following, we focus on the case in which two types of microbe are able to coexist at equilibrium.

**S2. The presence of the mutualism partner enhances the abundance of both microbes**

Now, let $\hat{A}$ be the equilibrium level of A in the system, including both type 1 and type 2 microbes, and let $A_{1}$ be the level of A in the system with type 1 and without type 2. We can prove $\hat{A}>A_{1}$ as follows:

Firstly, we note

$$\hat{A}\left[ k_{1}+r_{1}\frac{\hat{A}}{K_{1}+\hat{A}}\varphi_{1}\left( \hat{A} \right) \right]=\left( A_{T}-\hat{A} \right)\left[ k_{2}+r_{2}\frac{A_{T}-\hat{A}}{K_{2}+\left( A_{T}-\hat{A} \right)}\varphi_{2}\left( \hat{A} \right) \right]$$

(S9)

and

$A_{1}\left[ k_{1}+r_{1}\frac{A_{1}}{K_{1}+A_{1}}\varphi_{1}\left( A_{1} \right) \right]=\left( A_{T}-A_{1} \right)k_{2}$ . (S10)

Let $h\left( a \right)={a\left[ k_{1}+r_{1}\frac{a}{K_{1}+a}\varphi_{1}\left( a \right) \right]}/\left( A_{T}-a \right)$, which is a monotonically increasing function of $a$. Using this function, we can write

$h\left( \hat{A} \right)=k_{2}+r_{2}\frac{A_{T}-\hat{A}}{K_{2}+\left( A_{T}-\hat{A} \right)}\varphi_{2}\left( \hat{A} \right)>k_{2}=h\left( A_{1} \right)$ , (S11)

where we used that $\varphi_{2}\left( \hat{A} \right)$ is positive. Since $h\left( a \right)$ is monotonically increasing, we can conclude that $\hat{A}>A_{1}$, implying that the abundance of A is higher in the equilibrium with both type 1 and type 2 than in the equilibrium with only type 1. Note that the abundance of the type 1 microbe is $\hat{x}_{1}=\varphi_{1}\left( \hat{A} \right)$ in the presence of type 2 and $x_{1}=\varphi_{1}\left( A_{1} \right)$. Since function $\varphi_{1}\left( a \right)$ is monotonically increasing when it is positive, we can conclude that $\hat{x}_{1}>x_{1}$ from $\hat{A}>A_{1}$. Hence, the abundance of the type 1 microbe is enhanced by the presence of the type 2 microbe.

In a similar manner, we can prove that the abundance of the type 2 microbe is $\hat{x}_{2}$ in the presence of the type 1 microbe and is greater than $x_{2}$ in the absence of the type 1 microbe. The equilibrium abundance of A is $\hat{A}$ in the presence of both type 1 and type 2 microbes and is less than $\hat{A}$ in the presence of only the type 2 microbe, that is, $A_{2}$ ($\hat{A}$ < $A_{2}$). This is because the type 1 microbe consumes A, which is produced by the type 2 microbe.

**S3. Local stability of the candidate steady state**

We give the proof of the local stability of the steady state, first for the case in which all three variables are positive and then for the case in which one or more of the variables are zero.

*S3.1 When all three variables are positive at the focal steady state*

We first consider the case in which all three variables are positive at the focal steady state. The stability of a steady state can be established from the eigenvalues of the Jacobian matrix for the dynamics given by Eqs. (S1a–c) around the focal steady state. It is given as follows:

$J=\left( \begin{matrix} -\alpha& -\beta& \gamma\\ x_{1}F_{1}'(A) & \left( F_{1}\left( A \right)-m_{1}-{s_{1}x}_{1} \right)-{s_{1}x}_{1} & 0 \\ x_{2}F_{2}'(A) & 0 & \left( F_{2}\left( A \right)-m_{2}-{s_{1}x}_{2} \right)-{s_{1}x}_{2} \end{matrix} \right)$ (S12a)

where

$\alpha=-\frac{\partial}{\partial A}\frac{dA}{dt}=k_{1}+k_{2}+\frac{r_{1}K_{1}}{\left( K_{1}+A \right)^{2}}x_{1}+\frac{r_{2}K_{2}}{\left( K_{2}+A_{T}-A \right)^{2}}x_{2}>0$ , (S12b)

$\beta=-\frac{\partial}{\partial x_{1}}\frac{dA}{dt}=r_{1}\frac{A}{K_{1}+A}>0$ , (S12c)

$\gamma=\frac{\partial}{\partial x_{2}}\frac{dA}{dt}=r_{2}\frac{A_{T}-A}{K_{2}+(A_{T}-A)}>0$ , (S12d)

$F_{1}^{'}\left( A \right)=\frac{f_{1}}{K_{1}+A}\left\{ \frac{K_{1}}{K_{1}+A}\left( \gamma_{1}+RT\ln\frac{A}{A_{T}-A} \right)+\frac{RTA_{T}}{A_{T}-A} \right\}$ , (S12e)

$F_{2}^{'}\left( A \right)=-\frac{f_{2}}{K_{2}+A_{T}-A}\left\{ \frac{K_{2}}{K_{2}+A_{T}-A}\left( \gamma_{2}+RT\ln\frac{A_{T}-A}{A} \right)+\frac{RTA_{T}}{A} \right\}$ , (S12e)

and $x_{1}$, $x_{2}$ and *A* are the values in the steady state. The signs of $F_{1}^{'}\left( A \right)$ and $F_{2}^{'}\left( A \right)$ depend on the parameters as $\gamma_{1}$ and $\gamma_{2}$ can be both positive and negative. However, we can derive the following proposition concerning the sign of $F_{1}^{'}\left( A \right)$ and $F_{2}^{'}\left( A \right)$:

**Proposition 1**: $F_{1}^{'}\left( A \right)$ is positive when type 1 exists ($\hat{x}_{1}$ > 0 or $x_{1}$ > 0), and $F_{2}^{'}\left( A \right)$ is positive when type 2 exists ($\hat{x}_{2}$ > 0 or $x_{2}$ > 0).

The proof of this proposition is given as follows. From the condition of the existence of types 1 and 2 (*x*_1_ > 0 and *x*_2_ > 0 in the steady state),

$f_{1}\frac{A}{K_{1}+A}\left( \gamma_{1}+RTln\frac{A}{{(A}_{T}-A)} \right)-m_{1}>0$

$\gamma_{1}>\frac{m_{1}(K_{1}+A)}{f_{1}A}-RTln\frac{A}{{(A}_{T}-A)}$, (S13a)

$f_{2}\frac{A_{T}-A}{K_{2}+\left( A_{T}-A \right)}\left( \gamma_{2}+RTln\frac{{(A}_{T}-A)}{A} \right)-m_{2}>0$

$\gamma_{2}>\frac{m_{2}(K_{2}+(A_{T}-A))}{f_{1}(A_{T}-A)}-RTln\frac{{(A}_{T}-A)}{A}$ , (S13b)

where *A* is the value in the steady state. The derivatives of *F*_1_(*A*) and *F*_2_(*A*) with respect to *A* are

$F_{1}^{'}\left( A \right)=\frac{f_{1}}{K_{1}+A}\left\{ \frac{K_{1}}{K_{1}+A}\left( \gamma_{1}+RT\ln\frac{A}{A_{T}-A} \right)+\frac{RTA_{T}}{A_{T}-A} \right\}$ , (S14a)

$F_{2}^{'}\left( A \right)=-\frac{f_{2}}{K_{2}+A_{T}-A}\left\{ \frac{K_{2}}{K_{2}+A_{T}-A}\left( \gamma_{2}+RT\ln\frac{A_{T}-A}{A} \right)+\frac{RTA_{T}}{A} \right\}$ , (S14b)

$F_{1}^{'}\left( A \right)$ is positive when

$\gamma_{1}>\left( -\frac{RTA_{T}}{A_{T}-A}\frac{K_{1}+A}{K_{1}} \right)-RTln\frac{A}{{(A}_{T}-A)}$ . (S15a)

As the first term on the right-hand side of Eq. (S13a) is larger than that of Eq. (S15a), $F_{1}^{'}\left( A \right)$ is positive if Eq. (S13a) is satisfied or type 1 exists. $F_{2}^{'}\left( A \right)$ is negative when

$\gamma_{2}>\left( -\frac{RTA_{T}}{A}\frac{K_{2}+A_{T}-A}{K_{2}} \right)-RTln\frac{{(A}_{T}-A)}{A}$ . (S15b)

As the first term on the right-hand side of Eq. (S13b) is larger than that of Eq. (S15b), $F_{2}^{'}\left( A \right)$ is negative if Eq. (S15b) is satisfied.

Thus, we can conclude that $F_{1}^{'}\left( A \right)$ is positive when type 1 exists ($\hat{x}_{1}$ > 0 or $x_{1}$ > 0) and $F_{2}^{'}\left( A \right)$ is positive when type 2 exists ($\hat{x}_{2}$ > 0 or $x_{2}$ > 0).

(End of the proof of Proposition 1)

The Jacobian matrix around the focal steady state is

$J=\left( \begin{matrix} -\alpha& -\beta& \gamma\\ \delta_{1} & -\sigma_{1} & 0 \\ -\delta_{2} & 0 & -\sigma_{2} \end{matrix} \right)$, (S16)

where

$\sigma_{1}=s_{1}\hat{x}_{1}>0$ ,

$\sigma_{2}=s_{2}\hat{x}_{2}$>0 ,

$\delta_{1}=\frac{\partial}{\partial A}\frac{dx_{1}}{dt}=\hat{x}_{1}F_{1}'\left( \hat{A} \right)>0$ ,

$\delta_{2}=-\frac{\partial}{\partial A}\frac{dx_{2}}{dt}=-\hat{x}_{2}F_{2}'\left( \hat{A} \right)>0$ .

The characteristic polynomial for Eq. (S10) is

$\left( \lambda+\alpha\right)\left( \lambda+\sigma_{1} \right)\left( \lambda+\sigma_{2} \right)+\delta_{2}\gamma\left( \lambda+\sigma_{1} \right)+\delta_{1}\beta\left( \lambda+\sigma_{2} \right)=0$. (S17)

Let $\varphi\left( \lambda\right)=\left( \lambda+\alpha\right)\left( \lambda+\sigma_{1} \right)\left( \lambda+\sigma_{2} \right)+\delta_{2}\gamma\left( \lambda+\sigma_{1} \right)+\delta_{1}\beta\left( \lambda+\sigma_{2} \right)$,

$$\varphi\left( \lambda\right)=\lambda^{3}+\left[ \alpha+\sigma_{1}+\sigma_{2} \right]\lambda^{2}+\left[ \alpha\sigma_{1}+\alpha\sigma_{2}+\sigma_{1}\sigma_{2}+\delta_{2}\gamma+\delta_{1}\beta\right]\lambda$$

$+\left[ \alpha\sigma_{1}\sigma_{2}+\delta_{2}\gamma\sigma_{1}+\delta_{1}\beta\sigma_{2} \right]=0$ (S18)

By applying the Routh–Hurwitz criteria, all of the eigenvalues of Eq. (S18) have negative real parts because all of the coefficients are positive and the following inequality holds:

$\left[ \alpha+\sigma_{1}+\sigma_{2} \right]\left[ \alpha\sigma_{1}+\alpha\sigma_{2}+\sigma_{1}\sigma_{2}+\delta_{2}\gamma+\delta_{1}\beta\right]>\alpha\sigma_{1}\sigma_{2}+\delta_{2}\gamma\sigma_{1}+\delta_{1}\beta\sigma_{2}$ (S19)

because all of the terms on the right-hand side appear on the left-hand side of Eq. (S19) and the terms remaining on the left-hand side are all positive.

*S3.2 When one or both types of microbe are absent at the focal steady state*

Other cases include the situation in which one or both of the two microbe types are non-existent. For example, the steady state including $x_{1}=0$ is unstable if $F_{1}\left( A \right)-m_{1}>0$ because $x_{1}$ would increase when it was rare. By contrast, $x_{1}$ decreases with time if $F_{1}\left( A \right)-m_{1}<0$, and then, the stability is determined by examining whether the steady state is stable with respect to the remaining two variables ($x_{1}$ and $A$), which can be calculated from the Jacobian matrix of the dynamics with respect to these two variables.

If the focal steady state is $x_{1}>0$ and $x_{2}=0,$we have $\hat{x}_{1}=\frac{1}{s_{1}}\left( F_{1}\left( \hat{A}_{1} \right)-m_{1} \right)>0$ and $F_{2}\left( 0 \right)<m_{2}$. The characteristic polynomial at the candidate steady state is

$\left( F_{2}\left( \hat{A}_{1} \right)-m_{2}-\lambda\right)\left\{ \lambda^{2}-\left( \alpha+s_{1}\hat{x}_{1} \right)\lambda+(\alpha s_{1}\hat{x}_{1}+\hat{x}_{1}F_{1}'(\hat{A}_{1})\beta) \right\}=0.$ (S20)

The roots of the quadratic equation in the curly brackets are negative because $-\left( \alpha+s_{1}\hat{x}_{1} \right)<0$ and $\alpha s_{1}\hat{x}_{1}+\hat{x}_{1}F_{1}^{'}\left( \hat{A}_{1} \right)\beta>0$ (see Proposition 1). The Routh–Hurwitz condition shows that this steady state is locally stable.

If, instead, the focal steady state is $x_{1}=0$ and $x_{2}>0$, we can apply a similar argument with suffixes 1 and 2 switched.

If the focal steady state is the one with $x_{1}=x_{2}=0,$ then $F_{1}\left( A \right)-m_{1}<0$ and $F_{2}\left( A \right)-m_{2}<0$ are needed for stability because if the opposite inequality held, the corresponding variable would increase when it was small, indicating that the focal steady state was unstable. If both $F_{1}\left( A \right)-m_{1}<0$ and $F_{2}\left( A \right)-m_{2}<0$ hold, then the remaining one-dimensional dynamics are very simple: ${dA}/{dt}=-k_{1}A+k_{2}\left( A_{T}-A \right)$, which is obviously stable.

Hence, in short, the possible local stable steady state obtained by the method described in the text is always locally stable.

Hence, we can prove the local stability of the steady state that is the only candidate for the stable steady state. There is no other steady state that is locally stable. For a given set of parameters, only a single steady state satisfies the conditions for existence and local stability. We cannot prove the global stability of this state. However, according to our numerical analyses, we have never observed perpetual oscillation, chaotic fluctuation or bistability. This suggests that the locally stable steady state is, in fact, globally stable. If so, a numerical analysis of Eq. (4) confirmed that it is globally stable.

**S4. Niche expansion on the (**$\boldsymbol{B}_{\boldsymbol{e}}$**,** $\boldsymbol{C}$**)-plane**

In a similar manner to that in Section 3.3, the fundamental and realised niches of types 1 and 2 on the ($B_{e}$, $C$)-plane were numerically evaluated. On a plane where both axes are $\ln B_{e}$ and $lnC$, we again considered a parameter region with a square shape: $-3<\ln B_{e}<1$ and $-3<\ln C<1$. Then, we separated the squared region into $N_{f} \times N_{f}$ small squares ($N_{f}=51)$ of equal size ($4/51 \times4/51$). Niche size is measured by the number of small regions exhibiting a certain outcome to evaluate the prevalence of the result. See Section 3.3 for the definition of fundamental and realised niches of types 1 and 2. Here, the sizes of the realised and fundamental niches were calculated using the model presented by Eq. (4) (with the abundant resource premium (ARP) term) and the same model but without the ARP term (the model letting the second term on the right-hand side of Eqs. (5c) and (5d) be 0). The responses of niche sizes on the ($B_{e}$, $C$)-plane to $r_{2}$, $K_{2}$, $k_{2}$, $\Delta_{f}G_{C}^{o}$ and $\Delta_{f}G_{A_{e}}^{o}$are similar to those on the (*B*, $C_{e}$)-plane in Fig. 5 (Fig. S1). Overall, the difference between the realised niche and the fundamental niche on the ($B_{e}$, $C$)-plane was greater in a model with the ARP term included than in the corresponding model with the ARP term ignored, which suggests that the ARP encourages the expansion of the realised niches of types 1 and 2 caused by the presence of mutually beneficial metabolic partners.
