# Supplementary material for: Microbial material cycling, energetic constraints and ecosystem expansion in subsurface ecosystems
Source: Proc Biol Sci. 2020 Jul 29;287(1931):20200610. doi: 10.1098/rspb.2020.0610 (PMC7423649; doi:10.1098/rspb.2020.0610)
Supplement: Table S1 [file rspb20200610supp2.docx]

Table S1. Symbols, definition, units, default values and ranges of parameters

| Symbol | Definition | Unit | Default value |
| --- | --- | --- | --- |
| *x*_1_  *x*_2_  *A*  *A_e_*  *A_T_*  *B*  *B_e_*  *C*  *C_e_*  *k*_1_  *k*_2_  $q_{i}$  $c_{i}$  $r_{1}$  $r_{2}$  $K_{1}$  $K_{2}$  $K_{1B_{e}}$  $K_{2C}$  $\Delta_{f}G_{A}^{^{\circ}}$  $\Delta_{f}G_{A_{e}}^{^{\circ}}$  $\Delta_{f}G_{B}^{^{\circ}}$  $\Delta_{f}G_{B_{e}}^{^{\circ}}$  $\Delta_{f}G_{C}^{^{\circ}}$  $\Delta_{f}G_{C_{e}}^{^{\circ}}$  $-\Delta_{r}G_{1}^{^{\circ}}$  $-\Delta_{r}G_{2}^{^{\circ}}$  $R$  $T$  $m_{1}$  $m_{2}$  $s_{1}$  $s_{2}$ | Biomass of type 1 microbe  Biomass of type 2 microbe  Molar concentration of an electron-acceptor compound A  Molar concentration of an electron-donor compound A_e_  Sum of the molar concentration of A and A_e_  Molar concentration of an electron-acceptor compound of an element B  Molar concentration of an electron-donor compound of an element B  Molar concentration of an electron-acceptor compound of an element C  Molar concentration of an electron-donor compound of an element C  Abiotic reaction rate constant of Reaction 1  Abiotic reaction rate constant of Reaction 2  Amount of biomass that can be reproduced for a given energy gain of species *i*  Fraction of useful energy of species *i*  Maximum catalytic rate per unit of biomass of Type 1  Maximum catalytic rate per unit of biomass of Type 2  Michaelis–Menten constant for *A*  Michaelis–Menten constant for *A_e_*  Michaelis–Menten constant for *B_e_*  Michaelis–Menten constant for *C*  Standard Gibbs energy of formation of A  Standard Gibbs energy of formation of A_e_  Standard Gibbs energy of formation of B  Standard Gibbs energy of formation of B_e_  Standard Gibbs energy of formation of C  Standard Gibbs energy of formation of C_e_  Negative of standard Gibbs energy of Reaction 1  Negative of standard Gibbs energy of Reaction 2  Gas constant  Absolute temperature  Maintenance energy loss rate of type 1  Maintenance energy loss rate of type 2  Density-dependent mortality rate constant of type 1  Density-dependent mortality rate constant of type 2 | mg L^−1^  mg L^−1^  mmol L^−1^  mmol L^−1^  mmol L^−1^  mmol L^−1^  mmol L^−1^  mmol L^−1^  mmol L^−1^  mmol^−1^ L h^−1^  mmol^−1^ L h^−1^  mg kJ^−1^  no dimension  mmol h^−1^ mg^−1^  mmol h^−1^ mg^−1^  mmol L^−1^  mmol L^−1^  mmol L^−1^  mmol L^−1^  kJ mol^−1^  kJ mol^−1^  kJ mol^−1^  kJ mol^−1^  kJ mol^−1^  kJ mol^−1^  kJ mol^−1^  kJ mol^−1^  kJ mol^−1^ K^−1^  K  kJ h^−1^ mg^−1^  kJ h^−1^ mg^−1^  No dimension  No dimension | 1  *A_T_* − *A*  0.1  0.1  0.1  0.1  10^−6^  10^−5^  0.1  0.1  10^−0.5^  10^−0.6^  0.1  0.1  0.1  0.1  −8  −10  −15  −7  12  −10  See Eq. (5e)  See Eq. (5f)  8.314 × 10^−3^  288.15  10^−4^  10^−4^  1  1 |
