# Supplementary material for: Microbial material cycling, energetic constraints and ecosystem expansion in subsurface ecosystems
Source: Proc Biol Sci. 2020 Jul 29;287(1931):20200610. doi: 10.1098/rspb.2020.0610 (PMC7423649; doi:10.1098/rspb.2020.0610)
Supplement: Figure S1. [file rspb20200610supp3.pdf]

Figure S1

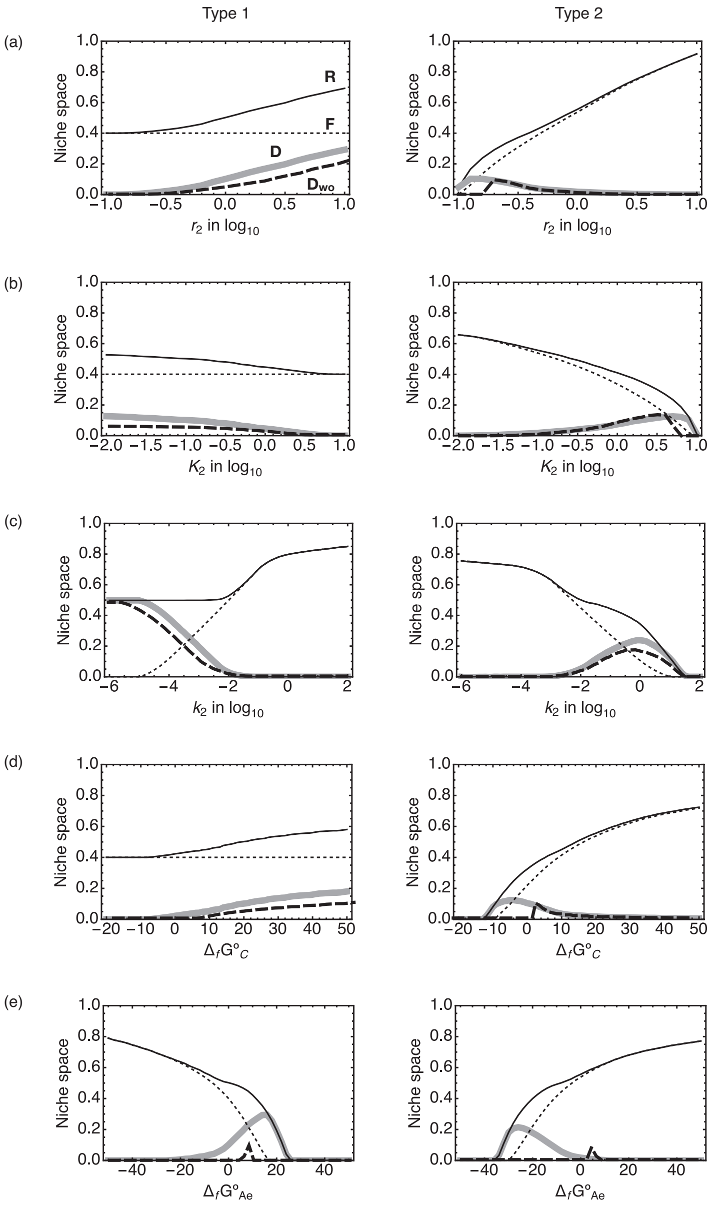

Figure S1: Parameter dependence of the size of the abundant resource premium (ARP)-driven niche space for type 1 (left panels) and type 2 (right panels). Niche sizes are measured by the number of sampled points on the $(\ln B_e, \ln C)$-plane, which is different from Fig. 5, where the size of the niche is defined by the area on the $(\ln B_e, \ln C)$-plane. Symbols and parameters are the same as in Fig. 5. The thick black dashed curve labelled by D_{wo} indicates the difference between the niche spaces of the realised niche and the fundamental niche calculated using a model ignoring the ARP term. $r_1 = 1$ and $r_2 = 1$. Other parameters are set to the default values shown in Table S1.
